# Monoclonal antibodies target conserved S1^B^ epitopes across deltacoronaviruses to inhibit porcine deltacoronavirus infection

**DOI:** 10.1128/mbio.01742-26

**Published:** 2026-08-13

**Authors:** Jiaru Zhou, Ran Jing, Mengdi Zhang, Changcheng Liu, Yiming Li, Hongmei Zhu, Wenjuan Du, Anan Jongkaewwattana, Berend Jan Bosch, Guiqing Peng, Qigai He, Mengjia Zhang, Wentao Li

**Affiliations:** 1 National Key Laboratory of Agricultural Microbiology, Hainan Research Institute, Hubei Hongshan Laboratory, College of Veterinary Medicine, Huazhong Agricultural University 124443, Wuhan, China; 2 College of Veterinary Medicine, Henan Agricultural University 731518, Zhengzhou, China; 3 Virology and Cell Technology Research Team, National Center for Genetic Engineering and Biotechnology (BIOTEC), National Science and Technology Development Agency (NSTDA) 61191, Klong Nueng, Thailand; 4 Virology Section, Infectious Diseases and Immunology Division, Department of Biomolecular Health Sciences, Faculty of Veterinary Medicine, Utrecht University 637796, Utrecht, the Netherlands; 5 Hubei Jiangxia Laboratory, Wuhan, China; The University of Iowa, Iowa City, Iowa, USA

**Keywords:** porcine deltacoronavirus（PDCoV）, monoclonal antibodies, virus neutralization, firefly luciferase reporter virus, cross-reactivity, embryonated chicken eggs

## Abstract

Porcine deltacoronavirus (PDCoV) is an emerging enteric pathogen that causes severe diarrhea, vomiting, and dehydration in piglets, and a recent human infection report raises concerns about its zoonotic potential. The S1 subunit of the PDCoV-spike protein is the primary target of neutralizing antibodies and is critical for viral attachment and entry. In this study, we generated and characterized eight monoclonal antibodies (mAbs) against S1, two targeting the S1^A^ domain, five targeting the S1^B^ domain, and one binding the S1^C^ domain. Using a genetically engineered luciferase reporter virus, we demonstrated that all five S1^B^-targeting mAbs neutralize viral infection by disrupting S1-aminopeptidase N receptor engagement. These S1^B^ mAbs cross-react with S1^B^ proteins from multiple human- and avian-origin deltacoronaviruses, suggesting the recognition of conserved antigenic regions. Importantly, we found that our PDCoV strain caused embryo lethality in embryonated chicken eggs (ECEs). Using this ECE lethal model, we performed a preliminary *in vivo* evaluation of the five S1^B^-specific neutralizing mAbs. All five mAbs improved embryo survival, with 3E9 providing complete protection against PDCoV-induced embryonic lethality. Collectively, these novel mAbs represent valuable tools for diagnosis and therapeutic development. Moreover, the luciferase-reporter virus and the ECE model establish robust platforms for future evaluation of neutralizing antibodies against PDCoV.

**IMPORTANCE:**

Porcine deltacoronavirus (PDCoV) is an emerging enteric coronavirus that threatens swine health and poses a potential risk of cross-species transmission; however, effective countermeasures remain limited. We generated and characterized a panel of monoclonal antibodies targeting the PDCoV S1 protein and identified five S1^B^-specific neutralizing antibodies that cross-react with S1^B^ proteins from multiple human- and avian-origin deltacoronavirusess. We also established complementary *in vitro* and preliminary *in vivo* platforms for antibody evaluation using a firefly luciferase reporter virus and an embryonated chicken egg model. Together, these findings expand the repertoire of PDCoV-neutralizing antibodies and provide practical tools for antibody characterization and preliminary protective efficacy assessment, facilitating future studies on antibody-based interventions against emerging deltacoronaviruses.

## INTRODUCTION

Porcine deltacoronavirus (PDCoV) is an emerging swine enteric coronavirus that causes acute watery diarrhea, vomiting, dehydration, and high mortality in suckling piglets, resulting in substantial losses to the swine industry (1–5). Notably, Haiti reported three pediatric cases of PDCoV infection, raising concerns about its zoonotic potential. Furthermore, PDCoV has demonstrated the ability to infect various other hosts, including chickens, calves, and mice, underscoring its broad host range and capacity for cross-species transmission (6–8).

The approximately 25.4 kb PDCoV genome encodes two large replicase polyproteins together with structural and accessory proteins required for virion assembly and infection (9). Coronavirus entry is mediated by the trimeric spike (S) glycoprotein. Its S1 subunit mediates attachment to host receptors and entry factors, and the S2 subunit drives membrane fusion (10, 11). The S1 subunit is further subdivided into four domains (S1^A^, S1^B^, S1^C^, and S1^D^). The S1^A^ domain binds sialylated glycans, facilitating the initial attachment of the virus to host cell surfaces, and the S1^B^ domain interacts with protein receptors to mediate viral entry into host cells (12–15). Aminopeptidase N (APN) has been identified as a functional receptor for PDCoV entry into host cells. The virus can utilize APN from various species, including pigs, humans, and chickens, indicating a broad host range and potential for interspecies transmission (16). The S1^C^ and S1^D^ domain likely correspond to structurally conserved domains analogous to the SD1/SD2 observed in SARS-CoV-2. These domains may contribute to maintaining the structural integrity of the S1-S2 interface, stabilizing the S trimer, and facilitating conformational rearrangements that precede membrane fusion (17).

Neutralizing monoclonal antibodies targeting the spike protein can block virus interaction with host cells, thereby inhibiting viral entry. The efficacy of neutralizing antibodies as therapeutic agents has been underscored by their pivotal role in combating the COVID-19 pandemic (18). In PDCoV research, progress has been made in the development of S protein-specific mAbs. PD33 and PD41, two humanized mouse-derived neutralizing antibodies, have demonstrated broad-spectrum antiviral activity by competitively blocking PDCoV-APN interactions (19). Du et al. generated and characterized a panel of neutralizing human mAbs targeting diverse epitopes on the S protein, improving our understanding of the antigenic landscape of PDCoV (20). The mAb 4A6 recognizes a conserved conformational epitope within the PDCoV S1 subunit, and in piglet challenge experiments, administration of 4A6 delayed the onset of diarrhea, alleviated disease severity, and reduced viral shedding (21). These advances reinforce the importance of developing and characterizing additional PDCoV mAbs to support improved diagnostic, therapeutic, and preventive strategies.

The use of firefly luciferase (Fluc)-based recombinant reporter viruses provides rapid, quantitative, and high-throughput platforms for evaluating neutralizing antibodies, vaccines, and antivirals. This approach has been widely validated in studies of SARS-CoV-2. Zettl et al. established a pseudovirus platform of vesicular stomatitis virus (VSV)*ΔG (FLuc) reporter virus harboring the SARS-CoV-2 spike protein that provides a novel and robust assay for rapid quantification of SARS-CoV-2-neutralizing antibodies in both convalescent individuals and vaccine recipients (22). Furthermore, Fluc-labeled pseudoviruses have been widely employed in clinical vaccine trials, including those involving the mRNA vaccines BNT162b2 and mRNA-1273, as well as the adenoviral vector vaccine Ad26.COV2.S, to evaluate cross-neutralization activity against emerging SARS-CoV-2 variants (23–25). Zhang et al. generated a recombinant PDCoV in which the accessory NS6 gene was replaced with green fluorescent protein (GFP), demonstrating that NS6 is dispensable for viral replication and can serve as a permissive site for reporter gene insertion (26). Building upon these findings, we engineered a recombinant reporter virus, rPDCoV-ΔNS6-Fluc, in which the NS6 gene was replaced with Fluc for quantitative assessment of the neutralizing activity of antibodies.

Neonatal piglets are the natural host and the gold-standard *in vivo* model for studies of PDCoV pathogenesis and therapeutic interventions. However, the high cost, specialized animal facilities, and intensive labor associated with piglet experiments limit their utility for the early-stage evaluation of candidate therapeutics. Genetic and evolutionary analyses indicate that PDCoV originated through multiple recombination events among avian deltacoronaviruses (27). In this context, embryonated chicken eggs (ECEs) represent an alternative experimental platform. Previous studies have demonstrated that PDCoV can infect ECEs and sustain serial passage following inoculation via the allantoic cavity, thereby enabling efficient viral propagation and isolation (7, 28). Here, we investigated the ability of our PDCoV strain to infect ECEs. Unexpectedly, infection resulted in embryonic lethality, indicating that this model may serve as a preliminary *in vivo* platform for assessing the protective activity of neutralizing antibodies.

In this study, we generated and characterized eight mAbs targeting the PDCoV S1 subunit and established a neutralization assay using a Fluc reporter virus for the rapid evaluation of neutralizing activity. Five S1^B^-specific mAbs exhibited neutralizing activity and recognized conserved antigenic regions shared among porcine, human, and avian deltacoronaviruses. In addition, these antibodies conferred protective efficacy in a newly established ECE challenge model. Collectively, these findings expand the understanding of conserved neutralizing epitopes within the PDCoV S1^B^ domain and provide useful experimental platforms and candidate antibodies for future studies of PDCoV and other emerging deltacoronaviruses.

## RESULTS

### Generation, purification, and characterization of eight monoclonal antibodies against the PDCoV S1 protein

**Fig 1:**
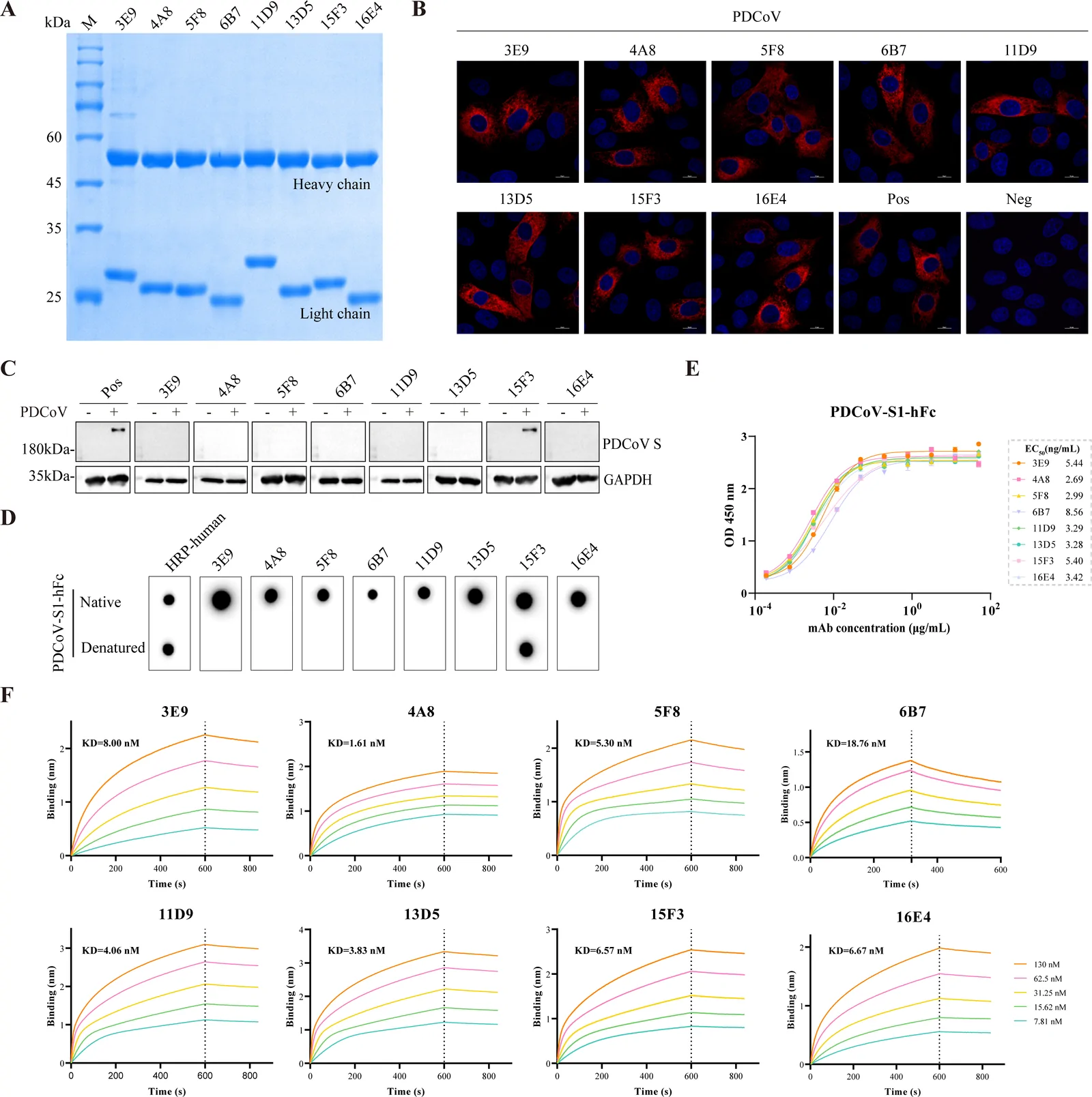
Generation, purification, and characterization of mAbs against PDCoV S1. (**A**) SDS-PAGE analysis of the purified mAbs. (**B**) IFA staining of PDCoV-infected pig kidney (LLC-PK1) cells using the generated mAbs as primary antibodies at a concentration of 1 µg/mL. Pos, sera from immunized mice. Neg, mock-infected LLC-PK1 cells. Scale bar, 10 µm. (**C**) Detection of the S protein in the lysates of PDCoV-infected Huh7 cells via western blot with the generated mAbs. Pos, and sera from immunized mice. (**D**) Dot blot analysis of mAb binding to recombinant PDCoV S1-hFc proteins under native and denatured conditions. (**E**) The binding of the mAbs to the recombinant PDCoV S1 protein was assessed via ELISA. The EC_50_ (half-maximal effective concentration) values were calculated using nonlinear regression analysis. (**F**) The kinetics of the binding of the mAbs to the recombinant PDCoV S1 protein were determined via BLI. The association and dissociation phases were analyzed, and the equilibrium dissociation constant (KD) values were calculated via Octet system analysis software.

The recombinant PDCoV S1 protein, fused with a mouse Fc tag and expressed in a HEK293F-based eukaryotic expression system, was used as the immunogen for antibody production. To generate mAbs against the PDCoV S1 protein, BALB/c mice were immunized with the recombinant PDCoV-S1-mFc protein following a prime-boost immunization strategy. Hybridoma cell lines were generated by fusing splenocytes from immunized mice with SP2/0 myeloma cells, followed by limited dilution cloning to obtain monoclonal hybridoma cells. Eight mAbs, designated 3E9, 4A8, 5F8, 6B7, 11D9, 13D5, 15F3, and 16E4, were successfully generated and purified from mouse ascites fluid. The purity of the obtained mAbs was evaluated by sodium dodecyl sulfate‒polyacrylamide gel electrophoresis (SDS-PAGE), which revealed distinct bands corresponding to the heavy chain and light chain, indicating successful purification (Fig. 1A). To characterize the binding properties of the generated mAbs, immunofluorescence assay (IFA), western blot, and dot blot assays were performed. All eight mAbs recognized the native S protein in PDCoV-infected cells and recombinant S1 protein, whereas only mAb 15F3 recognized the denatured S protein in both PDCoV-infected cell lysates and recombinant S1 protein (Fig. 1B through D; Fig. S1A). Furthermore, enzyme-linked immunosorbent assay (ELISA) was performed to assess the binding affinity of the eight generated mAbs against the S1 protein of PDCoV. All the mAbs exhibited concentration-dependent binding curves, with EC_50_ values ranging from 2.69 to 8.56 ng/mL (Fig. 1E). Kinetic measurements using biolayer interferometry (BLI) further confirmed high-affinity interactions, with the KD values ranging from 1.61 nM to 18.76 nM (Fig. 1F). Collectively, these results demonstrate that eight high-affinity monoclonal antibodies against the PDCoV S1 protein were successfully generated and characterized.

### Epitope mapping of the eight monoclonal antibodies

**Fig 2:**
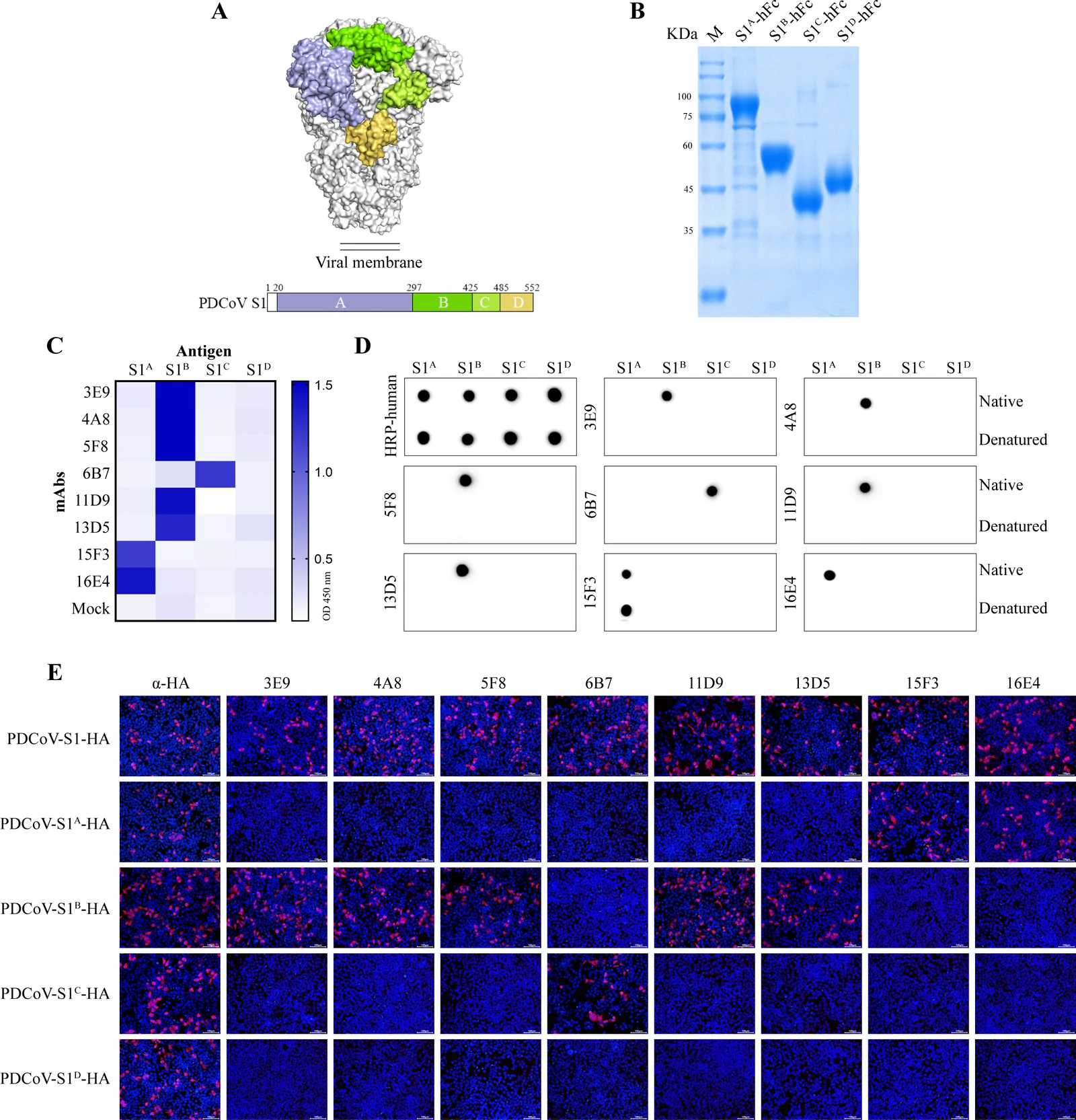
Epitope mapping of the mAbs. (**A**) Three-dimensional structure (PDB ID: 6B7N) and schematic representation of the PDCoV S protein showing differently colored S1^A^, S1^B^, S1^C^, and S1^D^ domains. (**B**) SDS-PAGE analysis of the domains (S1^A^, S1^B^, S1^C^, and S1^D^) of the PDCoV S1 subunit fused with the hFc tag expressed in HEK293F-based eukaryotic expression systems. (**C**) ELISA binding of mAbs to recombinant PDCoV S1^A^, S1^B^, S1^C^, and S1^D^ protein. (**D**) Dot blot analysis of mAbs binding to recombinant PDCoV S1^A^, S1^B^, S1^C^, and S1^D^ protein under native and denatured conditions. (**E**) IFA staining of HEK293T cells were transfected with plasmids expressing the S1^A^, S1^B^, S1^C^, or S1^D^ domains of PDCoV, and the eight generated mAbs were used to perform IFA. Scale bar, 100 µm.

The PDCoV S1 subunit comprises four domains: S1^A^, S1^B^, S1^C^, and S1^D^ (Fig. 2A). To determine the domain specificity of the generated mAbs, recombinant proteins corresponding to each domain fused with a human Fc tag were expressed in HEK293F cells. SDS-PAGE analysis confirmed the successful expression and purification of these recombinant proteins, as shown by clear bands of the expected sizes (Fig. 2B). ELISA and dot blot analyses using these recombinant proteins consistently showed that mAbs 15F3 and 16E4 specifically recognized the S1^A^ domain, mAbs 3E9, 4A8, 5F8, 11D9, and 13D5 recognized the S1^B^ domain, and mAb 6B7 recognized the S1^C^ domain, with no cross-reactivity observed among the four domains (Fig. 2C and D). Consistent with these results, IFA showed specific red fluorescence signals in pCAGGS-S1^A^-HA-transfected cells stained with mAbs 15F3 and 16E4; in pCAGGS-S1^B^-HA-transfected cells stained with mAbs 3E9, 4A8, 5F8, 11D9, and 13D5; and in pCAGGS-S1^C^-HA-transfected cells stained with mAb 6B7 (Fig. 2E). Together, these results show that the eight mAbs specifically recognize distinct domains of the PDCoV S1 subunit, defining their domain-level antigen recognition profiles.

### Identification of five S1^B^-specific neutralizing monoclonal antibodies using the reporter virus rPDCoV-ΔNS6-Fluc

**Fig 3:**
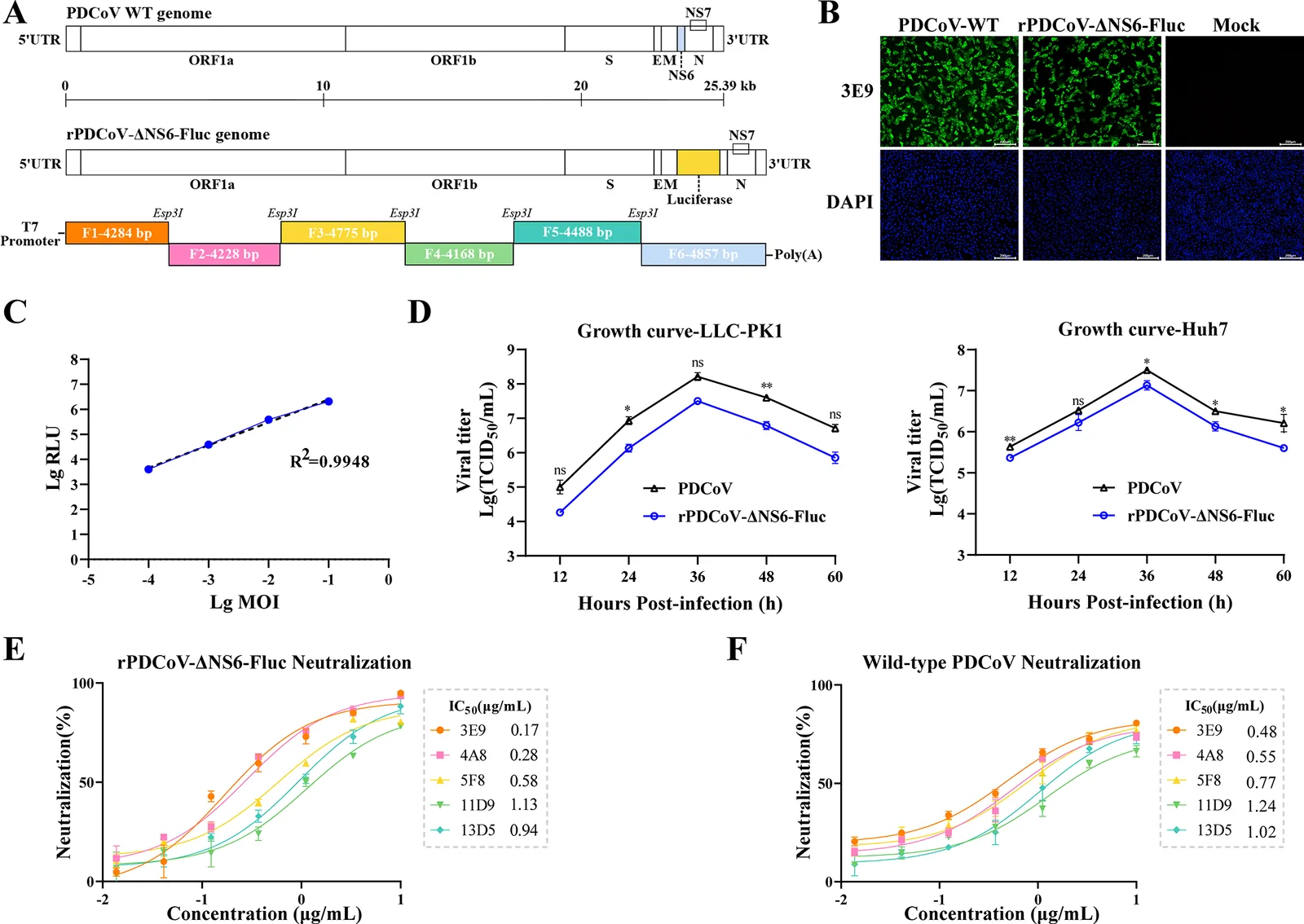
Construction of rPDCoV-ΔNS6-Fluc and evaluation of mAb neutralization activity in Huh7 cells. (**A**) Schematic representation of the genomes of wild-type PDCoV (PDCoV-WT) and the recombinant virus rPDCoV-ΔNS6-Fluc. (**B**) IFA staining of LLC-PK1 cells infected with PDCoV-WT or rPDCoV-ΔNS6-Fluc. Mock, mock-infected LLC-PK1 cells. Scale bar, 200 µm. (**C**) Correlation analysis between the luciferase activity of rPDCoV-ΔNS6-Fluc and the MOI. (**D**) Growth curves of PDCoV and rPDCoV-ΔNS6-Fluc in LLC-PK1 and Huh7 cells. (**E**) Determination of the neutralization activity of mAbs by rPDCoV-ΔNS6-Fluc in Huh7 cells. The virus neutralization rate was calculated by comparing the RLU values of the antibody-treated wells to those of the virus-only control wells. The IC_50_ was determined via nonlinear regression analysis in GraphPad Prism. (**F**) Validation of the neutralizing activities of mAbs by wild-type PDCoV in Huh7 cells. The virus neutralization rate was quantified by IFA staining, and IC_50_ values were determined via nonlinear regression analysis in GraphPad Prism.

To quantitatively assess the neutralization activity of the generated mAbs, we employed a recombinant PDCoV expressing Fluc (rPDCoV-ΔNS6-Fluc), which enables sensitive and high-throughput quantification of viral infection. Following the methodology described by Zhang et al. (26), the full-length genome of PDCoV was divided into six segments (F1‒F6). In segment F6, the NS6 gene was replaced with the Fluc gene to construct the reporter virus (Fig. 3A). The rescued virus was confirmed by IFA showing comparable S1 protein expression to wild-type PDCoV, indicating successful recovery (Fig. 3B). To evaluate the stability of the reporter virus, rPDCoV-ΔNS6-Fluc was serially passaged five times in LLC-PK1 cells. Luciferase activity and viral titers remained stable throughout serial passage (Fig. S2A), and sequence analysis confirmed that the Fluc insertion was maintained without detectable mutations (Fig. S2B), demonstrating the genetic stability of the reporter virus. LLC-PK1 cells were subsequently infected with rPDCoV-ΔNS6-Fluc at various multiplicities of infection (MOIs), and luciferase activity was quantified. The results revealed a strong linear correlation between the logarithm of the RLU and the logarithm of the MOI (R² = 0.9948, Fig. 3C), confirming that luciferase activity reflects the degree of viral replication. The reporter virus also exhibited replication kinetics comparable to those of wild-type PDCoV in both LLC-PK1 and Huh7 cells, with only a slight reduction in viral titer (Fig. 3D), which is consistent with previous findings indicating that NS6 is not necessary for viral replication.

The reporter virus was subsequently used to establish an FLuc-based neutralization assay for the rapid quantification of the neutralizing activity of the eight mAbs. The antibodies were preincubated with rPDCoV-ΔNS6-Fluc for 2 h before infecting Huh7 cells, which are naturally susceptible to PDCoV without trypsin treatment, thereby eliminating potential interference from exogenous enzymes in antibody binding. After 16 h of infection, luciferase activity was measured. The mAbs 3E9, 4A8, 5F8, 11D9, and 13D5, which target the PDCoV S1^B^ domain, exhibited dose-dependent neutralization activity, with 3E9 exhibiting the most potent effect (IC_50_: 0.17 μg/mL) (Fig. 3E). In contrast, mAbs 6B7, 15F3, and 16E4, which bind to the PDCoV S1^C^ or S1^A^ domains, displayed no neutralization activity (data not shown). To further validate the neutralization results obtained with the reporter virus assay, the five neutralizing mAbs were additionally evaluated against wild-type PDCoV using an immunofluorescence-based neutralization assay. All five antibodies retained neutralizing activity against wild-type PDCoV (Fig. 3F). Together, these results establish the reporter virus-based neutralization assay as a reliable platform for the identification and evaluation of PDCoV-neutralizing mAbs, through which five potent S1^B^-specific neutralizing antibodies were identified.

### S1^B^-specific neutralizing mAbs interfere with the interaction between PDCoV RBD and pAPN

**Fig 4:**
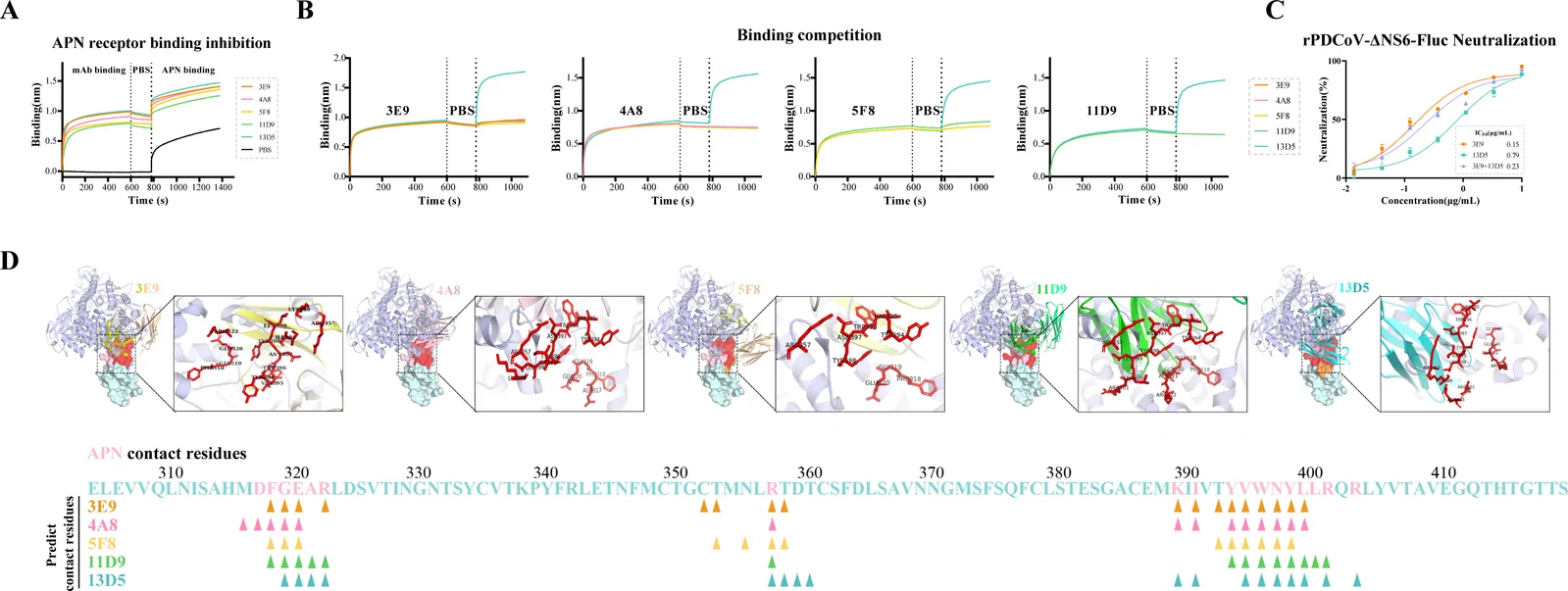
Mechanistic characterization of the S1B-specific neutralizing mAbs. (**A**) BLI-based assessment of APN receptor binding inhibition. (**B**) Antibody binding competition analyzed by BLI. (**C**) Neutralization assay evaluating the effect of combining mAbs 3E9 and 13D5. (**D**) Structural models of the antibody-RBD complexes generated using ABodyBuilder2 and HDOCK. The PDCoV RBD is shown in cyan, and pAPN is in purple according to the published PDCoV RBD-pAPN complex structure (PDB: 7VPP). Pink residues indicate previously reported pAPN-contact residues on the RBD, and orange residues indicate the predicted antibody-contact residues on the RBD. RBD residues predicted to be shared by the antibody-binding interface and the pAPN-binding interface are highlighted in red. The lower panel summarizes the overlap between the predicted antibody-contact residues and the reported pAPN-contact residues on the RBD.

To investigate the mechanism underlying neutralization, we first examined whether the neutralizing mAbs interfered with receptor binding. BLI analysis showed that all five S1^B^-specific mAbs interfered with the interaction between PDCoV S1 and porcine APN (pAPN) (Fig. 4A). Pairwise competition assays further demonstrated that mAbs 3E9, 4A8, 5F8, and 11D9 competed with one another for binding, whereas 13D5 exhibited a distinct competition profile (Fig. 4B). Because antibodies recognizing non-competing antigenic regions can potentially exhibit enhanced neutralizing activity when used in combination, we next evaluated a representative antibody cocktail consisting of mAbs 3E9 and 13D5. However, the combination did not exhibit enhanced neutralizing activity compared with 3E9 alone under the conditions tested (Fig. 4C).

To gain preliminary structural insight into the receptor-blocking activities of the neutralizing mAbs, antibody variable-domain structures were predicted using ABodyBuilder2, followed by molecular docking with the PDCoV receptor-binding domain (RBD) using HDOCK. The predicted antibody-contact residues were mapped onto the published PDCoV RBD-pAPN complex structure (PDB: 7VPP). Comparison with the reported pAPN-contact residues revealed partial overlap within the RBD, providing structural support for the receptor-blocking activities observed in the BLI assays (Fig. 4D). Together, the BLI results and preliminary structural predictions suggest that these neutralizing antibodies may interfere with the interaction between the PDCoV RBD and pAPN.

### Neutralizing mAbs exhibit cross-reactivity with human- and avian-origin deltacoronaviruses S1^B^ domains

**Fig 5:**
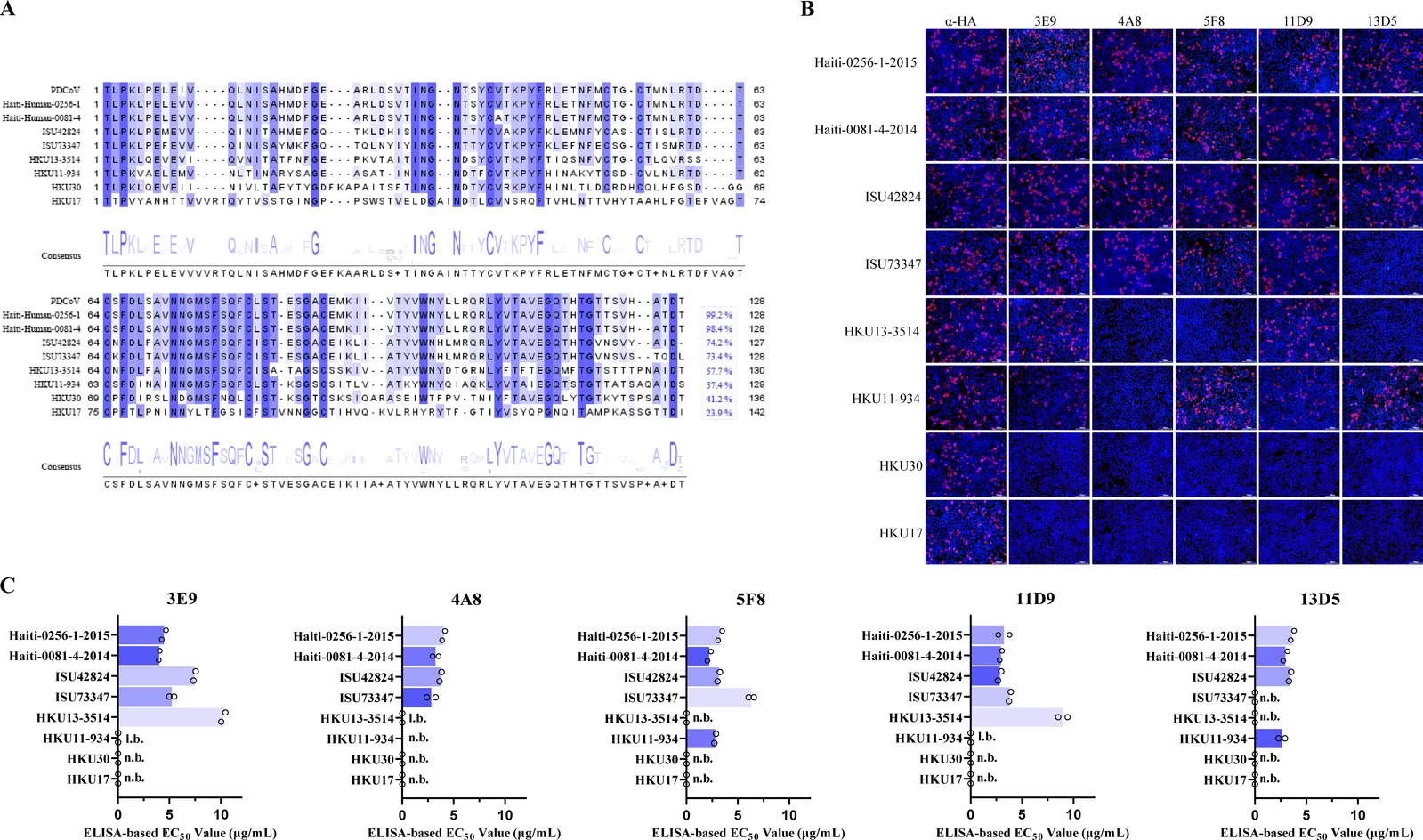
Cross-reactivity and binding affinity analysis of mAbs against the S1^B^ domains of multiple deltacoronaviruses. (**A**) Amino acid sequence alignment of the S1^B^ domain from PDCoV, Haiti-Human-0256-1-2015, Haiti-Human-0081-4-2014, ISU42824, ISU73347, HKU13-3514, HKU11-934, HKU30, and HKU17. (**B**) IFA staining of HEK293T cells transfected with plasmids encoding the S1^B^ domains of different deltacoronaviruses with HA tags. Scale bar, 100 µm. (**C**) ELISA-based determination of mAb binding to different deltacoronaviruses S1^B^ domains. The EC_50_ values are shown. l.b. indicates low binding, and n.b. indicates no binding.

To assess the cross-reactivity of the five neutralizing mAbs 3E9, 4A8, 5F8, 11D9, and 13D5, we evaluated the binding of these mAbs to S1^B^ proteins from both human- and avian-origin deltacoronaviruses via IFA and ELISA (Fig. 5B and C). All five mAbs exhibited cross-reactivity with these S1^B^ proteins, but none recognized HKU17 or HKU30. This observation is consistent with the amino acid sequence alignment (Fig. 5A), in which HKU17 and HKU30 displayed the lowest sequence identity with PDCoV, suggesting a lack of conserved epitopes recognized by the current antibodies. Interestingly, all five mAbs were able to bind effectively to the S1^B^ proteins of human-origin deltacoronaviruses, underscoring their potential as tools in human infections. 3E9 and 11D9 demonstrated the broadest reactivity, binding to all the deltacoronaviruses tested except HKU17 and HKU30, implying that they may target conserved and functionally critical epitopes within the S1^B^ domain. In contrast, 4A8, 5F8, and 13D5 exhibited limited binding profiles: 4A8 did not bind to HKU11, 5F8 failed to recognize HKU13, and 13D5 did not react with HKU13 or ISU73347. These distinct recognition patterns suggest that these mAbs may interact with more variable regions of the antigen. In conclusion, although none of the antibodies recognized HKU17 or HKU30, all five S1^B^-specific mAbs exhibited varying degrees of cross-reactivity with multiple deltacoronaviruses S1^B^ proteins, indicating that conserved antigenic epitopes are shared among these viruses.

### S1^A^-specific monoclonal antibodies lack hemagglutination-inhibitory activity against PDCoV

**Fig 6:**
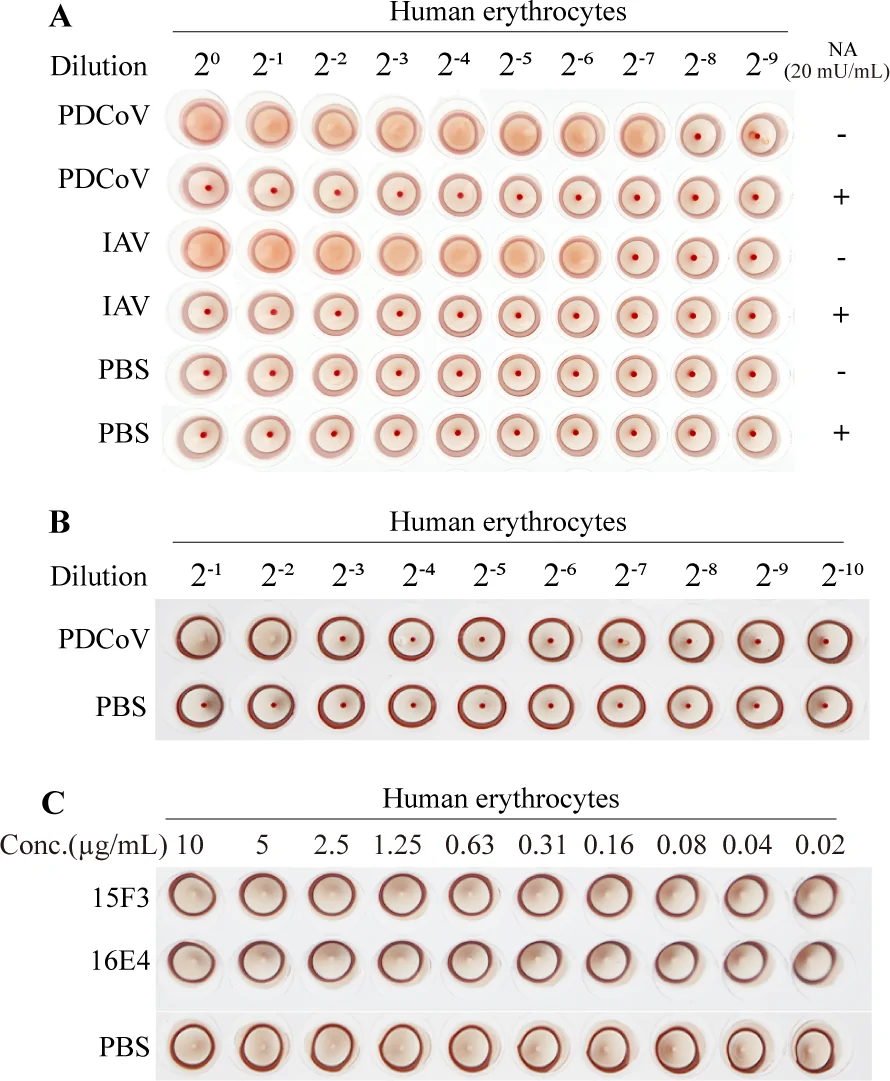
Evaluation of the hemagglutination inhibition activities of S1^A^-specific mAbs. (**A**) Identification of PDCoV hemagglutination activity via sialic acid binding. Neuraminidase-treated human erythrocytes were used to confirm that PDCoV-induced hemagglutination is dependent on sialic acid residues on the erythrocyte surface. (**B**) Determination of the hemagglutination titer of 25 μL PDCoV using human erythrocytes. (**C**) Hemagglutination inhibition assay evaluating the inhibitory effects of mAbs on PDCoV-induced hemagglutination.

The S1^A^ domain of PDCoV recognizes sialic acid residues on host cell surfaces, facilitating virus infection. This sialic acid-binding activity underlies the hemagglutination capability of PDCoV, in which the virus agglutinates erythrocytes via interactions with cell surface sialic acids. To assess whether the mAbs 15F3 and 16E4, which recognize the S1^A^ domain of PDCoV, can block its interaction with sialic acid and inhibit hemagglutination, we performed hemagglutination inhibition (HAI) assays. Initially, human erythrocytes were employed to confirm the hemagglutination capability of PDCoV. Treatment of erythrocytes with neuraminidase (NA), an enzyme that removes sialic acid residues, abolished PDCoV-induced hemagglutination, confirming the dependency of this activity on sialic acid binding (Fig. 6A). The virus was then standardized to 4 HA units in 25 μL for subsequent HAI assays (Fig. 6B). To validate the HAI assay, a previously characterized hemagglutination-inhibiting mAb 63 was included as a positive control and efficiently inhibited hemagglutination (29) (Fig. S3). Under the same conditions, neither mAb 15F3 nor mAb 16E4 inhibited PDCoV-induced hemagglutination (Fig. 6C), consistent with their lack of neutralizing activity.

### mAbs 3E9, 4A8, 5F8, 11D9, and 13D5 exhibit protective activity in PDCoV-challenged embryonated chicken egg model

**Fig 7:**
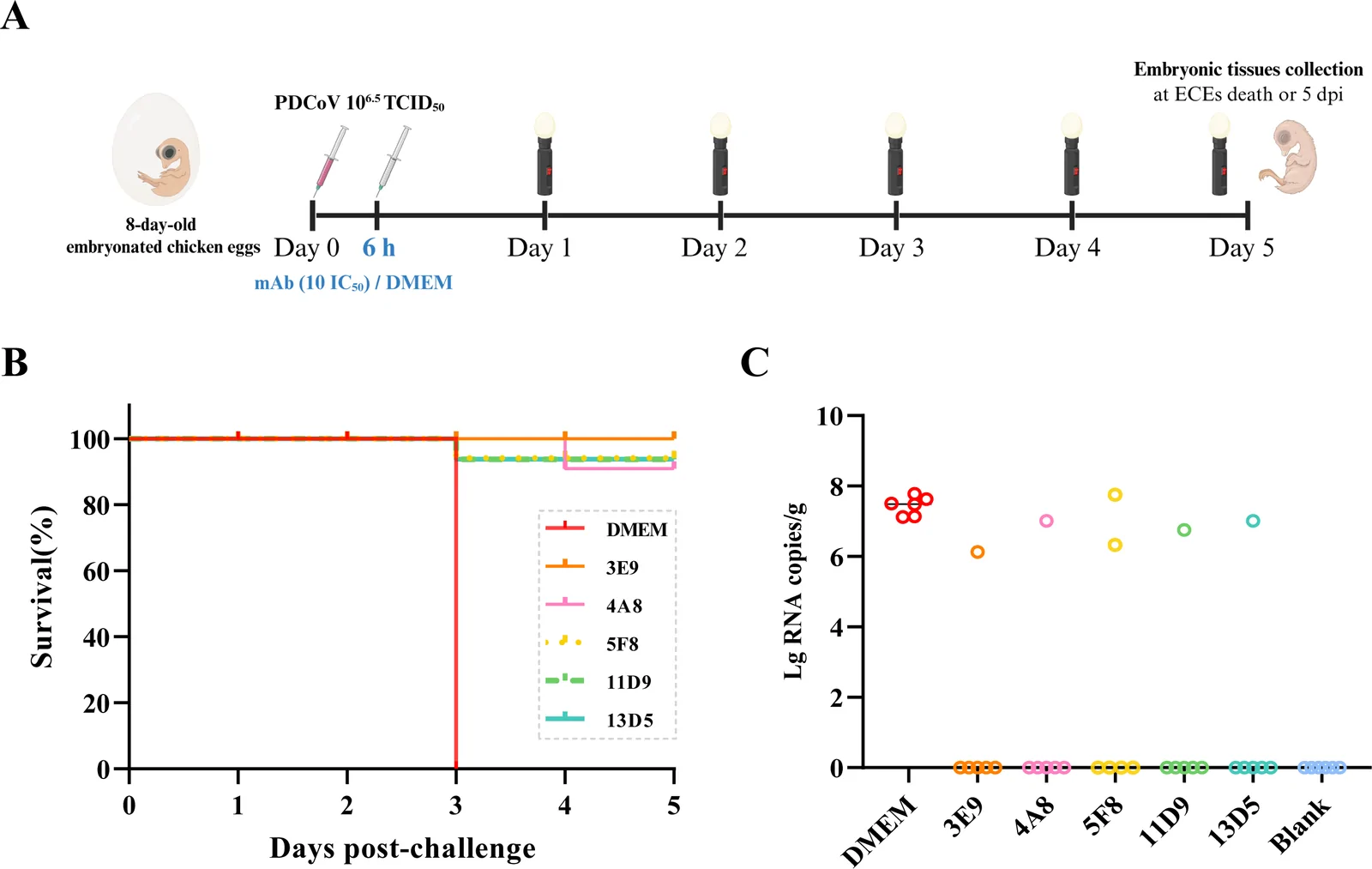
Protective activity of mAbs in PDCoV-challenged embryonated chicken eggs. (**A**) Experimental design for the evaluation of the protective activity of mAbs. Created in BioRender (Z. Jiaru, 2026, https://BioRender.com/ukhax0d). (**B**) Survival curves of mAb-treated, DMEM-mock-treated, and blank control groups. ECE survival was recorded daily for up to 5 days post-infection. (**C**) Viral load in embryonic tissues.

To extend our findings beyond *in vitro* assays, we performed a preliminary *in vivo* assessment using a lethal PDCoV challenge model in ECEs. Infection with 10^6.5^ TCID_50_ of PDCoV per egg resulted in complete embryonic mortality, providing a convenient platform for the preliminary evaluation of antibody efficacy. Antibody treatment was administered according to the scheme shown in Fig. 7A. All ECEs in the uninfected blank group survived, and no PDCoV RNA was detected in embryonic tissues (Fig. 7B and C). In contrast, ECEs in the infected, mock-treated group all succumbed by day 3 post-inoculation, and viral loads were detectable in their tissues. By contrast, the mAb-treated groups showed marked protection: the 3E9 group exhibited 100% survival; in the 4A8 group, there was only one death on day 4; and in the 5F8, 11D9, and 13D5 groups, each had one ECE die on day 3. Notably, substantial viral loads were detected in the tissues of all embryos that succumbed. Collectively, these results demonstrate that the five neutralizing mAbs confer protective activity in the PDCoV-challenged ECE model, supporting the utility of this model for the preliminary *in vivo* assessment of candidate therapeutic antibodies.

## DISCUSSION

In recent years, PDCoV has emerged as a serious threat to the global pig industry, notably by causing diarrhea outbreaks with high mortality in suckling piglets and leading to substantial economic losses. In addition to its impact on veterinary health, PDCoV has also raised growing public health concerns because of its ability to infect humans. The S1 subunit of the coronavirus spike protein, which mediates viral attachment to host cells, represents a key antigenic target for neutralizing mAbs. These mAbs, characterized by their high specificity, play crucial roles not only in diagnostic and therapeutic applications but also in advancing our understanding of pathogen-host interactions (30).

The S1^A^ domain of PDCoV interacts with sialylated glycans on the host cell surface, facilitating viral attachment prior to engagement with the primary receptor. Consistent with previous findings (31), our HA assays confirmed that PDCoV agglutinates human erythrocytes in a sialic acid-dependent manner. In our study, the mAbs 15F3 and 16E4 were identified as S1^A^-specific binding mAbs. Despite their specificity for the S1^A^ domain, neither antibody was able to inhibit PDCoV-induced hemagglutination. These findings indicate that binding of these antibodies is insufficient to interfere with the sialic acid-dependent attachment function of the S1^A^ domain. Notably, mAb 15F3 recognized denatured S protein in both western blot and dot blot assays, suggesting that it recognizes an epitope retained after protein denaturation, making it a useful tool for PDCoV antigen detection.

The spike S1^B^ domain of PDCoV is responsible for binding to host cell receptors, thereby facilitating viral entry. Previous studies have shown that antibodies targeting the S1^B^ domain can effectively block APN engagement and exhibit neutralization activity (20). To assess the neutralization activity of our generated mAbs efficiently and quantitatively, we constructed a recombinant virus, rPDCoV-ΔNS6-Fluc, which expresses Fluc via a reverse genetics system. The reporter virus maintained stable luciferase expression, viral replication, and sequence integrity during serial passage, demonstrating its phenotypic and genetic stability. Furthermore, rPDCoV-ΔNS6-Fluc exhibited replication kinetics comparable to those of wild-type PDCoV in both LLC-PK1 and Huh7 cells. Importantly, luciferase activity correlates well with viral titer (*R*² = 0.9948), allowing precise quantification of viral replication. Using rPDCoV-ΔNS6-Fluc, we developed a neutralization assay platform in Huh7 cells that supports PDCoV infection in the absence of trypsin, thereby eliminating the potential interference between trypsin and antibodies and enhancing assay reliability. Through this platform, we identified five S1^B^-specific mAbs 3E9, 4A8, 5F8, 11D9, and 13D5 with neutralizing activity. All five antibodies inhibited the interaction between PDCoV S1 and pAPN in the BLI assays, suggesting receptor blockade as a common feature associated with the neutralizing activity of the antibodies generated in this study. One possible explanation for this relatively uniform neutralization profile is the use of recombinant S1 as the immunogen. Unlike the native trimeric spike, recombinant S1 is presented in the absence of quaternary structural constraints, potentially increasing the accessibility of receptor-binding-associated epitopes and favoring the induction of receptor-blocking antibodies. Competition assays further showed that 13D5 belonged to a distinct competition group; however, combining 3E9 and 13D5 did not further enhance neutralizing activity, indicating that recognition of different antigenic regions alone was insufficient to improve the activity of this antibody combination. Structural modeling using ABodyBuilder2 and HDOCK further predicted partial overlap between the antibody-contact residues and previously reported pAPN-contact residues on the PDCoV RBD, providing preliminary structural support for the proposed receptor-blocking mechanism.

Given the avian origin of PDCoV (32) and its reported capacity to infect humans, we characterized the cross-reactivity of these five S1^B^-specific neutralizing mAbs across multiple deltacoronaviruses. Specifically, 3E9 and 11D9 exhibited broad cross-reactivity, recognizing all the tested deltacoronaviruses except HKU17 and HKU30. 4A8, 5F8, and 13D5 were also capable of binding to several of the tested deltacoronaviruses. Notably, these mAbs all demonstrated binding to the S1^B^ domains of human deltacoronaviruses, indicating that they recognize conserved antigenic epitopes. However, none of the mAbs recognized the S1^B^ domains of HKU17 or HKU30, consistent with our S1^B^ amino acid sequence identity analysis, which revealed only 41.2% and 23.9% identity for HKU30 and HKU17, respectively, as compared to PDCoV. Collectively, these findings highlight the potential utility of 3E9 and 11D9 as pan-deltacoronavirus detection antibodies. Furthermore, all five S1^B^-specific mAbs can be used for the detection of human-origin deltacoronavirus infections.

To extend our *in vitro* findings, we performed a preliminary *in vivo* assessment using a lethal ECE challenge model. In this model, all five neutralizing mAbs provided protection against PDCoV challenge, with mAb 3E9 conferring complete protection and the remaining four mAbs markedly delaying or preventing embryonic mortality. Although these findings support the protective activity of the antibodies *in vivo*, we acknowledge that the ECE model cannot fully mimic the pathogenesis, tissue tropism, or host immune responses of PDCoV infection in neonatal piglets, the natural host and gold-standard model for PDCoV infection. Therefore, the protective effects observed in the ECE model should be considered preliminary *in vivo* evidence. Nevertheless, the ECE model provides a rapid and cost-effective platform for the initial *in vivo* screening of candidate therapeutic antibodies before validation in neonatal piglets. Future studies in neonatal piglets will be needed to further evaluate the protective efficacy of these antibodies.

In conclusion, we generated eight mAbs with high affinity for different domains of the PDCoV S1 subunit, including S1^A^, S1^B^, and the first reported S1^C^-specific mAbs. Five S1^B^-specific mAbs exhibited neutralizing activity *in vitro* and protective activity in the ECE model, providing preliminary *in vivo* evidence of their therapeutic potential. These findings underscore the critical importance of the S1^B^ domain as an antigen for the induction of neutralizing antibodies. Moreover, the neutralization assessment platform based on Huh7 cells and the engineered rPDCoV-ΔNS6-Fluc enables high-throughput and sensitive evaluation, offering a practical approach for vaccine development and antiviral screening. At the same time, the ECE model may serve as a cost-effective and rapid preliminary *in vivo* model. Collectively, the neutralizing mAbs identified in this study, together with the reporter virus-based neutralization assay and the ECE challenge model, provide valuable tools for PDCoV research, therapeutic antibody development, and studies of emerging deltacoronaviruses.

## MATERIALS AND METHODS

### Cells and viruses

LLC-PK1 cells, human embryonic kidney 293T (HEK293T) cells, and human embryonic kidney 293F (HEK293F) cells were cultured in Dulbecco’s modified Eagle’s medium (DMEM; Gibco, USA) supplemented with 10% fetal bovine serum (FBS; ExCell Bio, China), 100 U/mL penicillin, and 100 μg/mL streptomycin (Gibco, USA) at 37°C with 5% CO_2_. Murine myeloma (SP2/0) cells were cultured in RPMI 1640 medium (Gibco, USA) supplemented with 20% FBS. The PDCoV strain CHN-HA-2017 (GenBank accession no. OR060947.1), PEDV strain GDU (GenBank accession no. KU985230.1), and TGEV strain WH-1 (GenBank accession no. HQ462571.1) were preserved in our laboratory. SADS-CoV strain GDS04 was kindly provided by Prof. Yongchang Chao (GenBank accession no. MF167434.1).

### Plasmid construction

The coding sequences for PDCoV S1 (residues 1–552), S1^A^ (residues 1–297), S1^B^ (residues 298–425), S1^C^ (residues 425–485), and S1^D^ (residues 486–552) were amplified, cloned, and inserted into the mammalian expression vectors pCAGGS or pcDNA3.1. For pCAGGS constructs, the Fc domain of either human or mouse IgG was fused at the C-terminus, resulting in the following constructs: pCAGGS-PDCoV-S1-mFc, pCAGGS-PDCoV-S1-hFc, pCAGGS-PDCoV-S1^A^-hFc, pCAGGS-PDCoV-S1^B^-hFc, pCAGGS-PDCoV-S1^C^-hFc, and pCAGGS-PDCoV-S1^D^-hFc. Similar cloning strategies were applied to generate S1^B^ constructs for deltacoronaviruses HKU11-934 (residues 302–530; GenBank accession no. YP_002308479.1), HKU13-3514 (residues 294–522; GenBank accession no. YP_002308506.1), HKU17 (residues 328–569; GenBank accession no. YP_005352846.1), HKU30 (residues 291–525; GenBank accession no. BBC54861.1), ISU42824 (residues 296–522; GenBank accession no. AWV67125.1), ISU73347 (residues 298–524; GenBank accession no. AWV67134.1), PDCoV Haiti-Human-0256-1-2015 (residues 298–525; GenBank accession no. MW685623.1), and PDCoV Haiti-Human-0081-4-2014 shared identical sequences with Haiti-Human-0329-4-201 (residues 297–524; GenBank accession no. MW685622.1; GenBank: MW685624.1).

### Expression and purification of recombinant proteins

HEK293F cells were seeded at a density of 1.1 × 10^6^ cells/mL in cell culture shaker flasks. The plasmids expressing recombinant protein and polyethyleneimine (PEI) were transfected into the cells at a ratio of 1:4. The cell supernatant was collected on the 5th day post-transfection, and the recombinant protein was subsequently purified via protein A Sepharose (Invitrogen, USA) and analyzed via SDS-PAGE.

### Generation of monoclonal antibodies targeting PDCoV S1

Eight-week-old female BALB/c mice were immunized with recombinant PDCoV-S1-mFc protein emulsified with Freund’s adjuvant (Merck, Germany) at 2-week intervals. Serum antibody levels were assessed via ELISA on day 7 after the third booster immunization. The mouse exhibiting the highest serum antibody titer received three additional booster immunizations at 3-day intervals before splenocyte fusion with SP2/0 myeloma cells. Hybridomas were screened via ELISA and IFA, subcloned three times, and expanded. Monoclonal antibodies were subsequently purified from mouse ascites fluid via protein A Sepharose beads according to the manufacturer’s instructions.

### Enzyme-linked immunosorbent assay

ELISA assays were utilized to evaluate the binding of the generated antibodies to the purified recombinant proteins. The purified recombinant proteins were coated onto 96-well NUNC Maxisorp plates (Thermo Fisher Scientific, USA) at a concentration of 100 ng/well and incubated overnight at 4°C. The plates were subsequently washed three times with phosphate-buffered saline containing 0.05% Tween-20 (PBST) and then blocked with 3% bovine serum albumin (BSA; Biosharp, China) in PBS containing 0.1% Tween-20 at room temperature for 2 h. The mouse serum, hybridoma supernatant, or purified antibodies were diluted to the corresponding concentrations in PBS containing 3% BSA and 0.1% Tween-20, added to each well, and incubated for 1 h at room temperature. After three washes, the plates were incubated for 1 h at room temperature with HRP-conjugated goat anti-mouse IgG (H + L) (Jackson ImmunoResearch, USA). The absorbance value was measured at OD 450 nm.

### Indirect immunofluorescence assay

The cells were washed twice with PBS and fixed with 3.7% paraformaldehyde (Merck, Germany) in PBS for 15 min at room temperature. The fixed cells were then washed three times with PBS and permeabilized with 0.1% Triton X-100 (Merck, Germany) for 12 min at room temperature. The cells were subsequently blocked in 3% BSA in PBS for 2 h at room temperature, followed by incubation with the primary antibody at a final concentration of 1 μg/mL overnight at 4°C. The cells were washed with PBST and incubated with Alexa Fluor 594-conjugated donkey anti-mouse IgG (H + L) (Jackson ImmunoResearch, USA) for 45 min at room temperature in the dark. The nuclei were stained with 4′,6-diamidino-2-phenylindole (DAPI, Solarbio, China), and fluorescence images were captured via an Olympus BX60 fluorescence microscope at 10× magnification.

### Western blot assay

Western blot (WB) assays were used to validate the expression of the recombinant proteins and identify whether the generated antibodies could recognize the linear epitope. The Huh7 and LLC-PK1 cells were infected with PDCoV and harvested at 16 h post-infection. The cells were lysed via RIPA lysis buffer (Beyotime, China) supplemented with protease inhibitors, and the lysates were denatured at 95°C for 10 min. The proteins in the lysate were separated via SDS-PAGE and subsequently transferred onto 0.45-μm polyvinylidene difluoride (PVDF) membranes (Millipore, USA) at 85 V for 120 min. The PVDF membranes were blocked with 5% nonfat milk at room temperature for 2 h and incubated with the generated antibodies diluted to 0.5 μg/mL in blocking buffer overnight at 4°C. HRP-conjugated goat anti-mouse IgG (H + L) (Jackson ImmunoResearch, USA) was used as the secondary antibody at a dilution of 1:5,000 for 1 h at room temperature, and the protein bands were visualized via a Tanon 5200 chemiluminescent imaging analysis system (Tanon, China).

### Dot blot assay

Purified recombinant PDCoV S1, S1^A^, S1^B^, S1^C^, and S1^D^ proteins were prepared in both native and denaturing conditions. For denaturation, proteins were mixed with SDS-loading buffer and heated at 95°C for 10 min, whereas native proteins were left untreated. Equal amounts of protein (1 μL per spot, containing 50 ng protein) were directly applied onto nitrocellulose membranes and air-dried. The membranes were blocked with 5% skim milk in TBST for 2 h at room temperature and incubated with the indicated monoclonal antibodies (1 μg/mL) for 2 h. After washing with TBST, membranes were incubated with HRP-conjugated goat anti-mouse IgG (1:5,000) for 1 h. Immunoreactive signals were detected using enhanced chemiluminescence (ECL) according to the manufacturer’s instructions.

### Antibody binding kinetics and affinity measurement using biolayer interferometry

BLI assays were used to evaluate the binding kinetics and affinity of the generated antibodies to the S1 subunit of PDCoV. The generated antibodies were loaded onto protein A biosensors (ForteBio, Germany) at a concentration of 44 nmol/L for 600 s. The biosensors were subsequently immersed in serial 2-fold dilutions of the recombinant PDCoV S1 protein, starting at a concentration of 130 nmol/L for association, followed by a dissociation phase in PBS. All the experiments were conducted at 30°C with constant shaking. The affinity constant was calculated via a 1:1 Langmuir binding model with ForteBio Data Analysis software.

### Construction and recovery of an infectious cDNA clone of PDCoV with the NS6 gene replaced by the firefly luciferase reporter gene

Following the strategy established by Zhang et al. (26), a recombinant PDCoV was generated in which the nonessential NS6 gene was replaced with the Fluc gene, resulting in a reporter virus designated rPDCoV-ΔNS6-Fluc. The full-length genome of PDCoV was divided into six overlapping fragments (F1–F6). Each fragment was amplified and cloned and inserted into pJET1.2/blunt vectors containing unique restriction sites recognized by the type IIS restriction enzyme Esp3I, enabling directional ligation. For the F6 fragment, the NS6 gene was replaced with the Fluc gene. The Fluc coding sequence was amplified via the following primers: Fluc-F: 5′-TGTATAAGTATATGTAatggaagacgccaaaacataaagaaagg-3′ and Fluc-R: 5′-GGTGTCAAAACTTTAcaatttggactttccgcccttcttg-3′. Following digestion with Esp3I, all six fragments were ligated via T4 DNA ligase (Thermo Fisher Scientific, USA) to assemble the full-length infectious clone of rPDCoV-ΔNS6-Fluc. *In vitro*-transcribed full-length RNA was synthesized via T7 RNA polymerase (Thermo Fisher Scientific, USA). In parallel, RNA encoding the PDCoV N protein was synthesized separately to facilitate virus rescue. The transcripts of the full-length complementary DNA (cDNA) and N gene were co-electroporated into LLC-PK1 cells via a Gene Pulser Xcell electroporation system (Bio-Rad, USA). After electroporation, cytopathic effects were monitored daily as evidence of successful virus recovery.

### Virus neutralization assay

We used rPDCoV-ΔNS6-Fluc and wild-type PDCoV to evaluate the neutralization activity of the mAbs. The mAbs were serially diluted 3-fold in DMEM, starting at a concentration of 10 µg/mL. For the antibody cocktail, mAbs 3E9 and 13D5 were mixed at a 1:1 ratio, with each antibody at 5 μg/mL (total antibody concentration, 10 μg/mL). Equal volumes of diluted mAbs and rPDCoV-ΔNS6-Fluc were mixed and incubated at room temperature for 2 h to allow efficient antibody-virus interactions. After incubation, the mixtures were added to Huh7 cells, which were then incubated at 37°C for 16 h. No exogenous trypsin was added during infection of Huh7 cells. For the luciferase-based assay, the culture supernatants were removed, and the cells were gently washed twice with PBS. Thirty microliters of cell passive lysis buffer (Promega, USA) were added, and the mixture was incubated at room temperature for 30 min to ensure complete cell lysis. Thirty microliters of luciferase assay reagent (Promega, USA) were added, and luminescence was immediately measured. Relative luminescence units (RLUs) were recorded. The virus neutralization rate was calculated by comparing the RLU values of the antibody-treated wells to those of the virus-only control. For wild-type PDCoV, infected cells were fixed with 3.7% paraformaldehyde and permeabilized with 0.1% Triton X-100. Cells were stained with mouse anti-PDCoV nucleocapsid antibody, followed by donkey anti-mouse Alexa Fluor 488-conjugated antibody. Images were captured using an Olympus BX60 fluorescence microscope at 4× magnification. The fluorescence intensity was analyzed using ImageJ software. The 50% inhibitory concentration (IC_50_) was determined via non-linear regression analysis using GraphPad Prism (version 9.5).

### APN receptor binding inhibition and antibody binding competition were performed using biolayer interferometry

APN receptor binding inhibition and antibody binding competition experiments were performed using biolayer interferometry (Octet Red348; ForteBio). Briefly, the PDCoV S1 protein was biotinylated using a Genemore G-MM-IGT Biotinylation Kit (Genemore, China) according to the manufacturer’s instructions. The biotinylated PDCoV S1 protein (10 μg/mL) was immobilized onto streptavidin (SA) biosensors for 10 min. Then, the biosensors were washed in PBS for 3 min. For assays, biosensors loaded with PDCoV S1 were first immersed in wells containing the primary monoclonal antibody (50 μg/mL) for 10 min to allow association. After a 3-min washing step in PBS, the sensors were then transferred to wells containing either the competing monoclonal antibody (50 μg/mL) or recombinant soluble porcine aminopeptidase N (pAPN; 150 μg/mL) for an additional association phase. The experiments were conducted at 30°C with constant shaking.

### Hemagglutination inhibition assay

The HAI assay was performed to evaluate the HAI activity of the generated antibodies against PDCoV S1^A^. First, the hemagglutination (HA) titer of PDCoV was determined, and the virus was diluted in PBS to a final HA titer of 160 HA units/mL. Serial 2-fold dilutions (25 μL) of the mAbs, starting at an initial concentration of 10 µg/mL, were mixed with an equal volume of the virus mixture and incubated at 4°C for 2 h. Subsequently, 50 μL of a 0.75% human erythrocyte suspension was added, and the mixture was further incubated at 4°C for 2 h. The HAI titer of each monoclonal antibody was defined as the lowest antibody concentration capable of inhibiting hemagglutination.

### Protective activity of mAbs in PDCoV-challenged embryonated chicken eggs

Eight-day-old specific pathogen-free ECEs were obtained from a certified supplier. Prior to inoculation, eggs were candled to verify embryo viability and normal development. For the mAb therapeutic experiment, eggs were randomly assigned into seven groups (*n* = 6 per group): five mAb treatment groups (mAbs 3E9, 4A8, 5F8, 11D9, and 13D5), one DMEM mock group, and one blank control (no treatment). First, except for the blank control group, eggs in the other groups were challenged with PDCoV at 10^6.5^ TCID_50_/egg (a dose predetermined to cause complete embryo lethality) via the allantoic cavity. Six hours post-infection, the eggs in the mAb treatment groups received intraallantoic inoculation of the respective mAbs (prepared at a concentration equal to 10 IC_50_). Eggs in the DMEM control group received an equivalent volume of DMEM. After injection, all eggs were incubated at 37°C, candled daily, and embryo survival was monitored. At embryo death or on day 5 post-infection, eggs were harvested and stored at 4°C for 12 h; embryonic tissues were then collected for subsequent analyses.

### Real-time reverse-transcription quantitative PCR analysis

Tissue samples from chicken embryos were processed for quantification of PDCoV RNA by real-time reverse-transcription quantitative PCR (RT-qPCR). Briefly, 1.0 g of embryonic tissue was homogenized in 1.0 mL of DMEM using magnetic-bead homogenization. The homogenate then underwent three freeze-thaw cycles at –80°C to disrupt the cells, followed by centrifugation to pellet debris. A 200 µL aliquot of the resulting supernatant was taken for downstream RNA extraction. Total viral RNA was extracted using the TRIzol reagent (Invitrogen, USA), following the manufacturer’s instructions. The extracted RNA was subsequently subjected to reverse transcription using the PrimeScript RT-Master Mix (Takara, Japan), according to the manufacturer’s protocol, to generate cDNA. qPCR was carried out using 2× Universal SYBR Green Fast qPCR Mix (ABclonal, China). The reactions were assembled following the supplier’s instructions, with the following primer pair targeting the N gene of PDCoV: Forward primer, 5′-AGCTCCCAAGCGGACTTTAC-3′; Reverse primer, 5′-AGGATCAGCCATACCCGTCT-3′. To generate a standard curve for absolute quantification, a recombinant plasmid containing the PDCoV N gene was serially diluted in 10-fold increments, covering a suitable dynamic range for quantification. The number of copies of PDCoV viral RNA in the tested samples was calculated based on the standard curve.

### Statistical analysis

Statistical analyses were performed using GraphPad Prism (version 9.5). Comparisons between the two groups were conducted using unpaired two-tailed Student’s *t*-tests. Data are presented as mean ± standard deviation (SD). A *P*-value of <0.05 was considered statistically significant. Statistical significance is indicated as follows: ns, not significant, *P* > 0.05, *, *P* < 0.05, **, *P* < 0.01, ***, *P* < 0.001, ****, *P* < 0.0001.

## SUPPLEMENTAL MATERIAL

Supplemental Figures mbio.01742-26-s0001.docx Figures S1-S3.

ASM does not own the copyrights to Supplemental Material that may be linked to, or accessed through, an article. The authors have granted ASM a non-exclusive, world-wide license to publish the Supplemental Material files. Please contact the corresponding author directly for reuse.
