# Supplementary material for: Monoclonal antibodies target conserved S1B epitopes across deltacoronaviruses to inhibit porcine deltacoronavirus infection
Source: mBio. 2026 Aug 13;17(9):e01742-26. doi: 10.1128/mbio.01742-26 (PMC13556247; doi:10.1128/mbio.01742-26)
Supplement: Supplementary file 1 [file mbio.01742-26-s0001.docx]

**Fig S1 Western blot validation of the reactivity of the generated mAbs in PDCoV-infected LLC-PK1 cells.** (A) Detection of the S protein in the lysates of PDCoV-infected LLC-PK1 cells via western blot with the generated mAbs. Pos, sera from immunized mice.

**Fig S2 Stability analysis of the recombinant reporter virus rPDCoV-ΔNS6-Fluc.** (A) Viral titers and luciferase activities of rPDCoV-ΔNS6-Fluc during five serial passages in LLC-PK1 cells. (B) Sequence alignment of the Fluc insertion region from passage 5 (P5) with the parental recombinant virus (P0).

**Fig S3 Validation of the hemagglutination inhibition assay using a positive control antibody.** (A) Determination of the hemagglutination titer of 25 μL GDU using human erythrocytes. (B) Hemagglutination inhibition assay using mAb 63, a previously characterized hemagglutination-inhibiting mAb, as the positive control. mAb 63 inhibited GDU-induced hemagglutination.
